# Preliminary Evaluations of [^11^C]Verubulin: Implications for Microtubule Imaging With PET

**DOI:** 10.3389/fnins.2021.725873

**Published:** 2021-09-08

**Authors:** Anton Lindberg, Andrew V. Mossine, Arturo Aliaga, Robert Hopewell, Gassan Massarweh, Pedro Rosa-Neto, Xia Shao, Vadim Bernard-Gauthier, Peter J. H. Scott, Neil Vasdev

**Affiliations:** ^1^Azrieli Centre for Neuro-Radiochemistry, Brain Health Imaging Centre, Centre for Addiction and Mental Health, Toronto, ON, Canada; ^2^Division of Nuclear Medicine, Department of Radiology, The University of Michigan Medical School, Ann Arbor, MI, United States; ^3^Translational Neuroimaging Laboratory, McGill Centre for Studies in Aging, Douglas Mental Health University Institute, Montreal, QC, Canada; ^4^McConnell Brain Imaging Centre, Montreal Neurological Institute, McGill University, Montreal, QC, Canada; ^5^Department of Psychiatry, University of Toronto, Toronto, ON, Canada; ^6^Division of Nuclear Medicine and Molecular Imaging, Department of Radiology, Massachusetts General Hospital, Harvard Medical School, Boston, MA, United States

**Keywords:** microtubules, PET, carbon-11, [^11^C]verubulin, [^11^C]HD-800, [^11^C]colchicine

## Abstract

[^11^C]Verubulin (a.k.a.[^11^C]MCP-6827), [^11^C]HD-800 and [^11^C]colchicine have been developed for imaging microtubules (MTs) with positron emission tomography (PET). The objective of this work was to conduct an *in vivo* comparison of [^11^C]verubulin for MT imaging in mouse and rat brain, as well as an *in vitro* study with this radiotracer in rodent and human Alzheimer’s Disease tissue. Our preliminary PET imaging studies of [^11^C]verubulin in rodents revealed contradictory results between mouse and rat brain uptake under pretreatment conditions. *In vitro* autoradiography with [^11^C]verubulin showed an unexpected higher uptake in AD patient tissue compared with healthy controls. We also conducted the first comparative *in vivo* PET imaging study with [^11^C]verubulin, [^11^C]HD-800 and [^11^C]colchicine in a non-human primate. [^11^C]Verubulin and [^11^C]HD-800 require pharmacokinetic modeling and quantification studies to understand the role of how these radiotracers bind to MTs before translation to human use.

## Introduction

Microtubules (MTs) consist of α- and β- tubulin dimers that polymerize to form the backbone of the neural cell structure and represent one of the most densely expressed proteins in the central nervous system (CNS) (Verhey and Gaertig, 2007; Janke, 2014; Goodson and Jonasson, 2018; Xia et al., 2020). The polymerization-depolymerization of tubulin is a dynamic process that allows the MT scaffold to change over time (Zhu et al., 2021). This process allows tubulin to exist as free and dimeric protein as well as polymerized MTs in a continuous cycle (Honore et al., 2005). The structure of MTs are different throughout the CNS (Dent, 2020). MTs in the white matter (WM) are more rigid and covered by myelin, whereas in gray matter (GM) the MTs have a more dynamic function and need to be able to polymerize and de-polymerize more frequently (Haroutunian et al., 2014; Wang and Young, 2014). Formation of MTs are a critical part of cell division and MT targeting therapeutics are successfully used in cancer treatment for inhibiting the rapid cell division associated with tumor growth (Jordan and Wilson, 2004). Loss of native functionality of MTs are a hallmark of several neurogenerative diseases such as Alzheimer’s disease (AD), Parkinson’s disease (PD), amyotrophic lateral sclerosis (ALS), and multiple sclerosis (MS) (Cappelletti et al., 2015; Clark et al., 2016; Sferra et al., 2020). Additionally, as tau is a MT associated protein, loss of MT functionality is closely linked with disease progression in non-AD tauopathies such as progressive supranuclear palsy (PSP), corticobasal degeneration (CBD), chronic traumatic encephalitis (CTE), and Pick’s disease (PiD) (Grijalvo-Perez and Litvan, 2014; Dubey et al., 2015; Calogero et al., 2018; Rogowski et al., 2021).

Imaging of MTs using positron emission tomography (PET) are much needed for diagnosis and drug discovery in neurodegenerative diseases. [^11^C]Paclitaxel, [^18^F]fluoropaclitaxel, and [^11^C]docetaxel are PET radioligands for MTs developed from existing pharmaceuticals. These PET radiotracers are considered to be MT stabilizing agents and bind to the taxane binding site, but all are substrates for efflux pumps such as P-glycoprotein (P-gp), making them unsuitable for translation as radiopharmaceuticals for imaging in the CNS (van der Veldt and Lammertsma, 2014). As such, there is interest in developing radiotracers that bind to the colchicine binding site which is another important pocket that can be targeted with tubulin polymerization destabilizers. [^11^C]Colchicine (labeled in the ring C-methoxy position) has been evaluated in preclinical PET imaging studies. However, as an efflux transporter substrate, [^11^C]colchicine is unsuitable for MT imaging in the CNS and has instead been used as a multiple drug resistance tracer and to confirm the indirect effect of P-gp action on colchicine-to-tubulin binding, as well as for imaging of peripheral tumors (Levchenko et al., 2000). Development of a new generation of MT-targeting PET radiotracers targeting the colchicine site has led to the development of [^11^C]verubulin (a.k.a.[^11^C]MCP-6827), [^11^C]HD-800 and [^11^C]WX-132-18B (Kumar et al., 2018; Sai et al., 2018, 2020). Molecules that bind to the colchicine site generally have simpler more hydrophilic structures and are more susceptible to blood-brain barrier (BBB) penetration than those targeting the taxane binding site, without inherent efflux issues (Lu et al., 2012). The colchicine site is also located on the intra-dimer face of β-tubulin making this binding site more accessible in un-polymerized or de-polymerized tubulin, in comparison to the taxol binding site which is located on the terminal end of the tubulin protein (Kaur et al., 2014).

Verubulin (Azixa, MCP-6827; IC_50_ = 1.5 nM to MT) is a drug in clinical trials that inhibits MT polymerization in tumor development and has shown to bind β-tubulin and disrupt dimerization (Chamberlain et al., 2014). [^11^C]Verubulin and [^11^C]HD-800 (IC_50_ = 4.3 nM to MT) have shown promise as PET radiotracers for MT imaging in mice (Kumar et al., 2018; Sai et al., 2018), and both have been used in baseline PET measurements in non-human primate (NHP) (Sai et al., 2019; Damuka et al., 2020).

**FIGURE 1:**
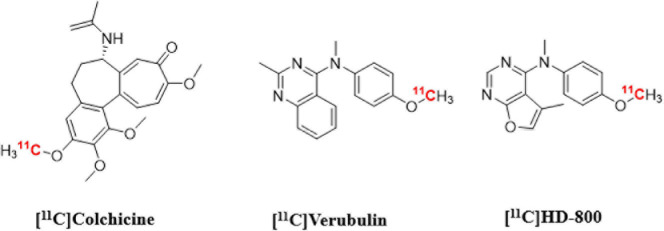
Chemical structures of colchicine-binding site PET radioligands evaluated in the present study.

Herein we present *in vitro* and *in vivo* results using [^11^C]verubulin for MT imaging in mouse in comparison with the first evaluation in rat, as well as an *in vitro* study with this radiotracer in human AD tissue. Our aim was to determine if [^11^C]verubulin binding is saturable *in vivo* in both mouse and rat in order to guide translational studies for higher species. Parallel to the evaluation of [^11^C]verubulin, we also conducted the first comparative *in vivo* PET imaging study with [^11^C]HD-800 and [^11^C]colchicine (labeled at the A-ring; Figure 1) in a NHP.

## Materials and Methods

### Radiochemistry

[^11^C]verubulin, [^11^C]HD-800 and [^11^C]colchicine (labeled at the A-ring; were labeled with carbon-11 by the same general method described below. Full experimental details for each radiotracer are available in the electronic supporting information (ESI).

Briefly, [^11^C]CO_2_ was reduced to [^11^C]CH_4_ and reacted with iodine to generate [^11^C]CH_3_I. The latter was subsequently converted to [^11^C]CH_3_OTf by passing through a AgOTf-on-silica column. [^11^C]CH_3_OTf was bubbled through the reaction mixture (1 mg precursor + 5 μL 1M methanolic TBAOH in 100 μL anhydrous DMF) at room temperature for 3 min. HPLC mobile phase (1 mL) was added and the reaction mixture was loaded onto a Phenomenex Luna C18, 10 μm, 10 × 250 mm semi-preparative HPLC column and eluted using 50% CH_3_CN in 10 mM NH_4_OAc_aq_ at 5 mL/min. The radioactive product was collected into a flask containing 50 mL of water, then passed through a Waters C18 1-cc vac-cartridge. The product was eluted with 500 μL dehydrated ethanol into an intermediary vial, then rinsed with 4.5 mL sterile buffered saline. Quality control and molar activity (*A*_m_) was measured using a Phenomenex Luna C18, 5 μm, 4.6 × 150 mm analytical HPLC column using 60% CH_3_CN in 10 mM NH_4_OAc_aq_ at 1 mL/min. Identity of the radiochemical product was confirmed via co-injection of standard of the desired product.

### *In vitro* Autoradiography

Anesthesia was induced in the rat using 5% isoflurane (2–4 L/min). The rat was decapitated and the brain was removed. The brain was immediately frozen in a container with isopentane cooled to −40°C on dry ice for approximately 4 min, then transferred to a −80°C freezer for long-term storage.

Postmortem brain samples from patients with an antemortem positive diagnosis Consortium to Establish a Registry for Alzheimer’s Disease (CERAD) (Janke, 2014) and CN (CERAD negative) subjects were obtained from the Douglas-Bell Canada Brain Bank with the approval of the scientific journal of the Brain Bank and the research ethics boards of the Douglas Institute. A total of seven AD patients (74 ± 10 years-old, Braak stage 6, tau positive) and seven CN individuals (72 ± 11 years-old, Braak stage 0, tau negative) were studied. The AD and CN samples had the cerebellum, hippocampus, and pre-frontal regions assessed (one AD individual and one CN individual did not have viable hippocampal sections). The postmortem time ranged from 8.5 to 18.25 h and 17.25 to 21.25 h in patients with AD and individuals with CN, respectively.

For *in vitro* autoradiography studies, the frozen tissues were cut into 20 mm thick sections and thawed on coated microscope slides using a frozen slide microtome (Leica CM3050 S) at −15°C. The samples were then dried at room temperature for 20–30 min and preincubated in phosphate buffered saline (pH 7.4) for 10 min to remove endogenous ligands. The samples were dried again in ambient air for 20–30 min and subsequently incubated in phosphate buffered saline (PBS) (pH = 7.6) with [^11^C]verubulin @ 234 GBq/M (single batch). For the rat, 3.89 MBq [^11^C]verubulin was used adding 50 mL (reference) and 50 mL (blocker @ 500 μM) for the blocking experiments. Human autoradiography was conducted with 28.49 MBq [^11^C] verubulin in 600 ml of buffered saline solution phosphate. In all experiments, the incubation time was 40 min. After the incubation, the tissues were dipped three times in the phosphate buffered solution and, subsequently dipped in distilled water at 4°C and dried under a stream of fresh air.

Finally, tissues were exposed to a radioluminographic imaging plates (Fujifilm BA SMS2025) for 240 min and scanned with an Amersham Typhoon scanner (50 μM isotropic). The activity in photo-stimulated luminescence units per mm^2^ was calculated using Image-Gauge 4.0 software (Fujifilm Medical Systems, Inc.).

Region of interests in rat follow previously described methods for radiotracer validations (Parent et al., 2013). For human tissue, activity in the brain region of each individual was measured in three equidistant regions of interest (ROI) placed by an experimenter blind to clinical diagnosis. Then, an average value of these three ROIs was used as the absorption of the final region.

### PET Imaging

Rodent and non-human primate PET imaging studies were performed in accordance with the standards set by the Institutional Animal Care and Use Committee (IACUC) at the University of Michigan. Rodent imaging was done using Sprague Dawley rat (female, 4–5 months old, ∼320 g) or mouse (female, 4–6 weeks old, ∼24 g). NHP imaging was done using a mature female rhesus monkey (body weight ∼9.0 kg with negligible variation throughout the duration of the study, 15 years of age). PET imaging was conducted using a Concorde Microsystems MicroPET P4 tomograph. For rodent imaging, animals were anesthetized (isoflurane), and positioned in the PET scanner. Anesthesia was maintained with 2–4% isoflurane/O_2_ throughout the imaging studies. In baseline PET measurements, following a transmission scan, the animal was injected (i.v. tail vein injection) with the radiotracer as a bolus over 1 min. Amounts [^11^C]verubulin injected were 21.2 MBq (0.575 mCi) in rat and 13.3 MBq (0.360 mCi) in mouse. In pretreatment PET measurements the animal was injected (i.v. tail vein injection) with verubulin (5 mg/kg) 20 min prior to radiotracer. Amounts [^11^C]verubulin injected were 17.9 MBq (0.486 mCi) in rat and 18.1 MBq (0.491 mCi) in mouse. The brain was imaged for 90 min (4 × 1 min frames—1 × 1.75 min frames—1 × 2.5 min frames—1 × 3.75 min frames—1 × 5 min frames—1 × 7.5 min frames—6 × 10 min frames).

For NHP imaging, the animal was anesthetized in its home cage (ketamine) and transported to the imaging suite where it was intubated and the animal positioned on the bed of the PET gantry. A head-holder was used to prevent motion artifacts. Isoflurane anesthesia was then applied (2–4% isoflurane/O_2_) and continued throughout the study. A venous catheter was inserted into one hindlimbs and, following a transmission scan, the animal was injected *i.v.* with [^11^C]colchicine (182 MBq, 4.937 mCi), [^11^C]verubulin (149 MBq, 4.045 mCi) or [^11^C]HD-800 (181 MBq, 4.906 mCi) as a bolus over 1 min, and the brain imaged for 90 min (4 × 1 min frames −1 × 1.75 min frames −1 × 2.5 min frames −1 × 3.75 min frames −1 × 5 min frames −1 × 7.5 min frames −6 × 10 min frames).

Following the scans, emission data were corrected for attenuation and scatter and reconstructed using the 3D maximum *a priori* method (3D MAP algorithm). Using a summed image of the entire data set, regions of interest (ROIs) were drawn manually on multiple planes to obtain volumetric ROIs for the whole brain, white matter, thalamus, cortex, and cerebellum. The volumetric ROIs were then applied to the full dynamic data sets to obtain the regional tissue time-radioactivity data.

### Statistical Analysis

Statistical significance (*p*-values) was determined using one-way ANOVA.

## Results

### Radiochemistry

[^11^C]Verubulin was radiolabeled by *O*-[^11^C]methylation using [^11^C]CH_3_OTf according to a previously published method (Kumar et al., 2020). The product was obtained in 1.5 ± 0.6% radiochemical yield (non-decay corrected (NDC), *n* = 4), with > 99% radiochemical purity and molar activity of 204 ± 52 GBq/μmol (5.5 ± 1.4 Ci/μmol). [^11^C]HD-800 was similarly radiolabeled in 1.2 ± 0.7% (NDC, *n* = 3) radiochemical yield, with > 99% radiochemical purity and molar activity 159 ± 100 GBq/μmol (4.3 ± 2.7 Ci/μmol). [^11^C]Colchicine was also labeled by the same method in 0.5% radiochemical yield (NDC, *n* = 1) with > 99% radiochemical purity and molar activity of 389 GBq/μmol (10.5 Ci/μmol, see ESI).

### PET Imaging of [^11^C]Verubulin in Rodent

**FIGURE 2:**
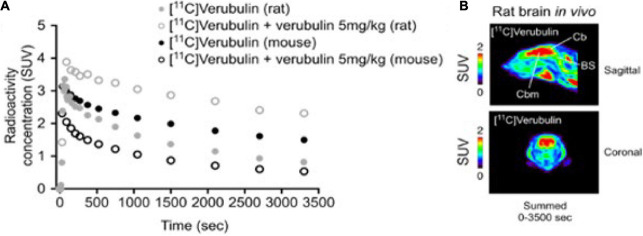
**(A)** Whole brain time-activity curves of [^11^C]Verubulin in rodent (mouse and rat; *n* = 1). **(B)** *In vivo* PET imaging of [^11^C]Verubulin in rat (baseline, summed images).

PET imaging in mouse (*n* = 1) using [^11^C]verubulin showed an initial uptake in whole brain of 3.2 SUV followed by a steady decrease to below 2 SUV at the end of the 70 min PET scan. In blocking experiments using verubulin (5 mg/kg), a significant decrease in radioactivity in brain over 70 min compared to baseline measurements (Figure 2).

PET imaging in rat (*n* = 1) using [^11^C]verubulin showed an initial uptake in whole brain of 3.3 SUV followed by a steady decrease to below 2 SUV at the end of the 70 min PET measurement. In blocking experiments using verubulin (5 mg/kg), an increase of radioactivity in brain was observed. The initial uptake of radioactivity in whole brain was 4.0 SUV and remained higher than in baseline measurements during the full 70 min PET measurement (Figure 2).

### *In vitro* Characterization of [^11^C]Verubulin in Rat and Human Tissues Using Autoradiography

**FIGURE 3:**
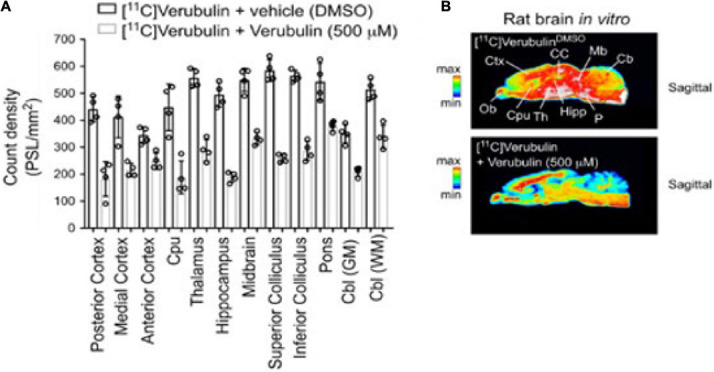
Preclinical characterization of [^11^C]verubulin in rat brain tissues. **(A)** Regional quantification for the *in vitro* autoradiography of [^11^C]verubulin in the normal rat brain (*n* = 4) Comparison between baseline and verubulin co-treatment. **(B)** Autoradiography images of rat brain showing distribution of [^11^C]verubulin at baseline (top) and with co-treatment of verubulin (bottom).

Slices of brain tissue from rat (*n* = 4) were used in autoradiography experiments. Baseline measurements using [^11^C]verubulin showed significantly higher radioactivity in all regions compared to blocking measurements using verubulin (500 μM) (posterior cortex, *p* < 0.0001; medial cortex, *p* < 0.0001; anterior cortex, *p* = 0.0328; caudate putamen (Cpu), *p* < 0.0001; thalamus, *p* < 0.0001; hippocampus, *p* < 0.0001; midbrain, *p* < 0.0001; superior colliculus *p* < 0.0001; inferior colliculus, *p* < 0.0001; pons *p* < 0.0001; cerebellum (Cbl) GM, *p* = 0.0003; Cbl WM, *p* < 0.0001) (Figure 3).

**FIGURE 4:**
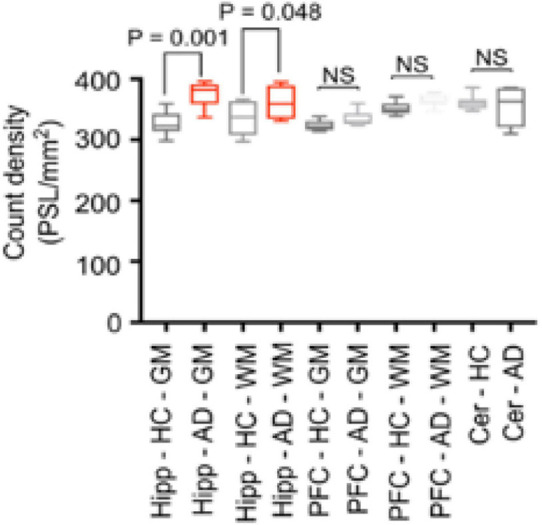
Preclinical characterization of [^11^C]verubulin in human brain tissues. Comparison of total binding of [^11^C]verubulin in GM and WM in hippocampus, prefrontal cortex and cerebellum from HC (*n* = 7) and AD (*n* = 7) tissue.

[^11^C]verubulin had a significantly higher binding in GM and WM in AD hippocampus (*p* < 0.001, *p* < 0.048, respectively) in autoradiography experiments using human tissue from healthy controls (*n* = 7) and human AD tissue (*n* = 7). In prefrontal cortex (PFC) and cerebellum tissue there was no significant difference in [^11^C]verubulin binding between AD and healthy control (Figure 4). In brain tissue from healthy controls [^11^C]verubulin showed higher uptake in WM than in GM.

Potential off-target binding of verubulin was evaluated against 371 CNS active kinases without any significant interactions, using a commercial assay (Eurofins; full data in ESI).

### Preliminary Neuro-PET Imaging to Compare [^11^C]Colchicine, [^11^C]Verubulin, and [^11^C]HD-800 in NHP at Baseline

**FIGURE 5:**
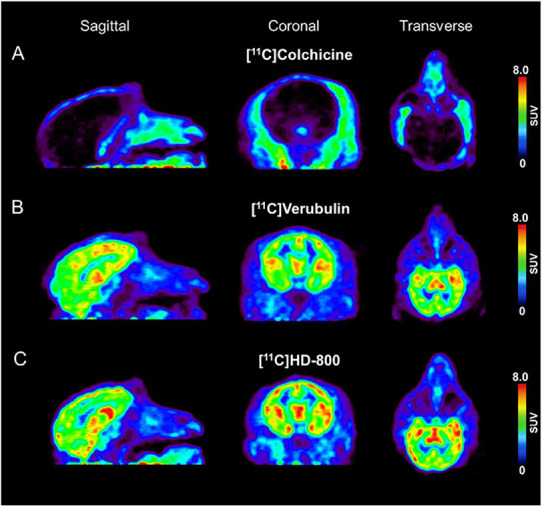
Baseline PET imaging (SUV images summed 6–60 min) depicting sagittal, coronal, transverse views in Rhesus monkey for: **(A)** [^11^C]colchicine **(B)** [^11^C]verubulin and **(C)** [^11^C]HD-800.

**FIGURE 6:**
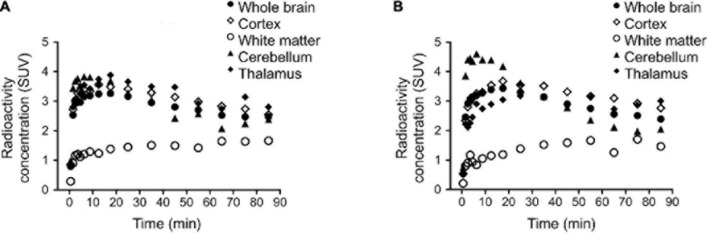
Regional brain time activity curves (TACs) in rhesus monkey for: **(A)** [^11^C]verubulin and **(B)** [^11^C]HD-800. TAC for [^11^C]colchicine not shown due to low brain penetration.

A single rhesus monkey was used in baseline PET studies comparing [^11^C]colchicine, [^11^C]verubulin, and [^11^C]HD-800 (*n* = 1 for each compound). In the first study, [^11^C]colchicine (182.7 MBq, 4.937 mCi) was administered intravenously and radioactivity reached a maximum of 0.3 standard uptake value (SUV) in whole brain after 3 min, decreased to 0.03 SUV after 6 min and increased back to 0.2 SUV after 90 min, and is possibly attributed to formation of a brain penetrating radiometabolite (Figure 5A). In a second study, [^11^C]verubulin (149.7 MBq, 4.045 mCi) was administered intravenously and radioactivity reached a maximum SUV of 3.2 in whole brain within 5 min and decreased to 2.5 SUV after 90 min (Figures 5B, 6A). Low levels of radioactivity were detected in WM (<2 SUV) during the duration of the scan. In a final study [^11^C]HD-800 (181.5 MBq, 4.906 mCi) was administered intravenously and radioactivity reached a maximum SUV of 3.4 in whole brain after 20 min and decreased to 2.3 SUV after 90 min (Figures 5C, 6B). Higher uptake was initially detected in cerebellum (SUV = 4.3 after 3 min) but SUV decreased to 2 after 90 min. Low radioactivity was detected in WM (SUV < 2) for the duration of the scan.

## Discussion

### PET Imaging in Rodents

[^11^C]verubulin showed initial brain uptake and decrease in baseline PET measurements in both mouse and rat, consistent with previously reported data in mice (Kumar et al., 2018, 2020). The ability to block tubulin binding in mouse has been shown previously using [^11^C]WX-132-18B and [^11^C]HD-800 (Sai et al., 2018, 2020), therefore the decreased radiotracer uptake of [^11^C]verubulin in mouse is consistent with our expectations. On the contrary, an increase in [^11^C]verubulin brain uptake was observed in rat when co-treated with verubulin as the blocking agent. The contradictory results from the blocking experiments in mouse and rat with [^11^C]verubulin is the first evidence of species differences with this radiotracer to our knowledge. This observation may also extend to other radiotracers that target the colchicine binding site, albeit, there are several possible explanations for this observed increase in radioactivity in the CNS following pre-treatment of verubulin (i.e., peripheral effects, radiometabolism, saturation of efflux proteins, effect on the MT polymerization kinetics, etc.) and additional studies are needed to confirm our preliminary results in rat. Nonetheless these species dependent results suggest a potential issue for using [^11^C]verubulin or any other colchicine binding site PET tracers to quantify MT in higher species and should be taken into consideration before planning future studies.

### *In vitro* Characterization of [^11^C]Verubulin in Rat and Human Tissues Using Autoradiography

In light of the conflicting *in vivo* PET data in rats with [^11^C]verubulin, further *in vitro* characterization was carried out to evaluate [^11^C]verubulin in rat and human tissue using autoradiography. As predicted, co-treatment with verubulin (500 μM) showed a decrease in binding in all regions in the autoradiography studies. As the increase in brain uptake observed in the *in vivo* rat PET measurements following [^11^C]verubulin administration under pretreatment conditions was not replicated in our autoradiography study, the rationale for the increased uptake *in vivo* requires further investigation.

In baseline autoradiography experiments in human tissue, [^11^C]verubulin shows no significant difference in binding between WM and GM in either HC and AD tissue. In autoradiography studies comparing [^11^C]verubulin binding in brain tissue from AD patients with brain tissue from HC, significantly higher binding of [^11^C]verubulin in AD hippocampus brain tissue was observed. We initially hypothesized that MT degradation in AD pathology would result in lower MT density and consequently decreased binding of [^11^C]verubulin. This assumption depends on clearance of free tubulin from the CNS in AD pathology. Without tubulin being cleared from the brain, MT degradation could result in higher concentration of free tubulin, which is in line with our preliminary results. Since significant differences in radiotracer binding could only be quantified in hippocampus, and although indications of increased [^11^C]verubulin uptake was seen in PFC as well, a better model of MT dynamics in AD pathology is needed in order to apply the colchicine binding site PET tracers for MT quantification in AD.

### Preliminary Neuro-PET Imaging to Compare [^11^C]Colchicine, [^11^C]Verubulin, and [^11^C]HD-800 in NHP

Preliminary PET imaging studies in NHP of [^11^C]verubulin (Damuka et al., 2020) was recently reported along with [^11^C]HD-800 as a meeting abstract (Sai et al., 2019), concurrent with our ongoing study. Herein we report the first comparative *in vivo* PET imaging study of [^11^C]colchicine, [^11^C]verubulin, and [^11^C]HD-800 in a NHP. All three radiotracers were evaluated in baseline PET measurements in NHP. The lack of brain uptake in NHP by [^11^C]colchicine was expected as colchicine is a known P-gp substrate in rodents, and was considered in the present work to explore a potential species difference as this radiotracer was previously labeled in a different position (C-ring vs. A-ring) and imaged only in rodents (Levchenko et al., 2000). Both [^11^C]verubulin and [^11^C]HD-800 show initial uptake in brain with no signs of efflux issues that follows previously reported PET studies with MT imaging agents in NHP (Kumar et al., 2019; Sai et al., 2019; Damuka et al., 2020). The PET summation images of [^11^C]verubulin and [^11^C]HD-800 show a heterogenous distribution between WM and GM in brain. Additionally, the binding kinetics in WM are markedly slower than in whole brain and does not reach a distinctive maximum radioactivity uptake during the 90 min scan time. The difference in binding kinetics in WM compared to GM, could be explained by the higher degree of myelination around the MT structure in WM, protecting the colchicine binding site from access by the radiotracers and making the kinetics of the ligand binding slower. Conversely, in GM the MT structure is more flexible, with more polymerization and de-polymerization resulting in greater access to the intra-dimer colchicine binding site. Nonetheless, [^11^C]verubulin and [^11^C]HD-800 require additional studies that include but are not limited to pharmacokinetic modeling with arterial input functions and radiometabolite analysis as well as studies to confirm saturability and specificity for MTs before translation to human use.

## Conclusion

Our preliminary PET imaging studies of [^11^C]verubulin in rodents suggests species differences in brain uptake between mouse and rat under pretreatment conditions. In mouse, the expected decrease in brain uptake of the radiotracer occurred, while in rat an increase in overall brain uptake was observed when pretreated with verubulin. *In vitro* autoradiography with [^11^C]verubulin in rat brain tissues showed the expected decrease in binding under blocking conditions, albeit human brain tissue revealed an unexpected higher uptake of [^11^C]verubulin in AD patient tissue compared with HC. Both [^11^C]verubulin and [^11^C]HD-800 were able to penetrate the BBB with fast clearance in NHP brain, and as expected [^11^C]colchicine did not enter the brain in higher species. Further studies to confirm saturable and specific binding of [^11^C]verubulin or [^11^C]HD-800, as well as if these radiotracers can be used for quantification of MT density in neurodegenerative disorders remains to be determined prior to human translation.

## Data Availability Statement

The raw data supporting the conclusions of this article will be made available by the authors, without undue reservation.

## Ethics Statement

The rodent and non-human primate PET imaging studies were reviewed and approved by the Institutional Animal Care and Use Committee (IACUC) at the University of Michigan.

## Author Contributions

VB-G, PS, and NV designed research. VB-G, AM, AA, XS, and RH performed research. VB-G and GM contributed new reagents and analytical tools. AL, VB-G, AM, PR-N, PS, and NV analyzed the data. AL, VB-G, and NV wrote the manuscript. All authors reviewed the manuscript.

## Conflict of Interest

The authors declare that the research was conducted in the absence of any commercial or financial relationships that could be construed as a potential conflict of interest.

## Publisher’s Note

All claims expressed in this article are solely those of the authors and do not necessarily represent those of their affiliated organizations, or those of the publisher, the editors and the reviewers. Any product that may be evaluated in this article, or claim that may be made by its manufacturer, is not guaranteed or endorsed by the publisher.
