# Supplementary material for: Preliminary Evaluations of [11C]Verubulin: Implications for Microtubule Imaging With PET
Source: Front Neurosci. 2021 Sep 8;15:725873. doi: 10.3389/fnins.2021.725873 (PMC8456034; doi:10.3389/fnins.2021.725873)
Supplement: Supplementary file 1 [file Table_1.DOCX]

Supplementary Material

Preliminary evaluation of [^11^C]verubulin reveals species differences: Implications for microtubule imaging with PET

Anton Lindberg^†^, Andrew V. Mossine^†^, Arturo Aliaga, Robert Hopewell, Gassan Massarweh, Pedro Rosa-Neto, Xia Shao, Vadim Bernard-Gauthier^*^, Peter J. H. Scott^*^ and Neil Vasdev^*^

***Co-Corresponding Authors**: Vadim Bernard-Gauthier ([vadimbgauthier@gmail.com](mailto:vadimbgauthier@gmail.com)); Peter J.H. Scott ([pjhscott@umich.edu](mailto:pjhscott@umich.edu)) and Neil Vasdev ([neil.vasdev@utoronto.ca](mailto:neil.vasdev@utoronto.ca))

# Index

Radiochemistry procedures 2.

Kinase activity data 8.

**[^11^C]Verubulin**

HPLC conditions:

Semiprep column: Phenomenex Luna C18, 10 micron, 10x250 mm

Semiprep eluent: 50% MeCN 10mM NH4OAc pH:7.3 5mL/min

Analytical column: Phenomenex Luna C18, 5 micron, 4.6x150 mm, 1 mL/min

Analytical eluent: 60% MeCN 10mM NH4OAc pH:7.4 5mL/min

Delivered 30 minute beam (approx. 3 Ci [^11^C]-CO_2_) to hot cell 2. [^11^C]-CO_2_ was converted to [^11^C]-methyl iodide using standard methods; approx. 660 mCi [^11^C]-methyl iodide was produced, which was converted to [^11^C]-methyl triflate by passing gaseous through a AgOTf-on-silica column.

[^11^C]-methyl triflate was bubbled through the reactor medium (1mg precursor + 5µL 1M methanolic TBAOH in 100 µL anh. DMF) at room temperature for 3 minutes at 15 mL/min with He carrier gas. Semipreparative eluent (1 mL, see above) was added to the reactor and contents of reactor were loaded onto semiprep column. Collected peak from 15:20 - 16:15 minutes into 50mL dilution water, then passed this mixture through a Waters C18 1cc vac cartridge (pre-activated with 10 mL ethanol, then 10 mL water). Eluted product with 500 µL dehydrated ethanol into an intermediary vial, then rinsed with 4.5 mL sterile buffered saline. Passed the formulated product solution through a sterile filter and into a 10 mL product vial. Reformulated product activity: 34.7 mCi (rat), 57.7 mCi (NHP). Specific activity: 5714 Ci/mmol (rat), 6676 Ci/mmol (NHP).

QC results: >99% RCP, product identity confirmed by coinjection with genuine standard.


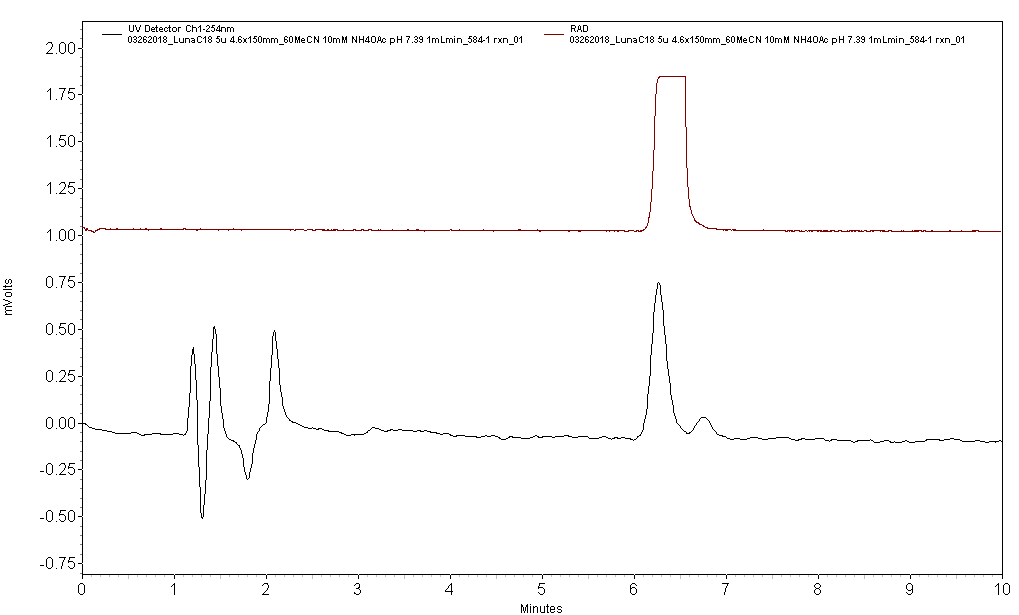


[^11^C]verubulin QC HPLC traces (Rad above, 254 nm UV below)


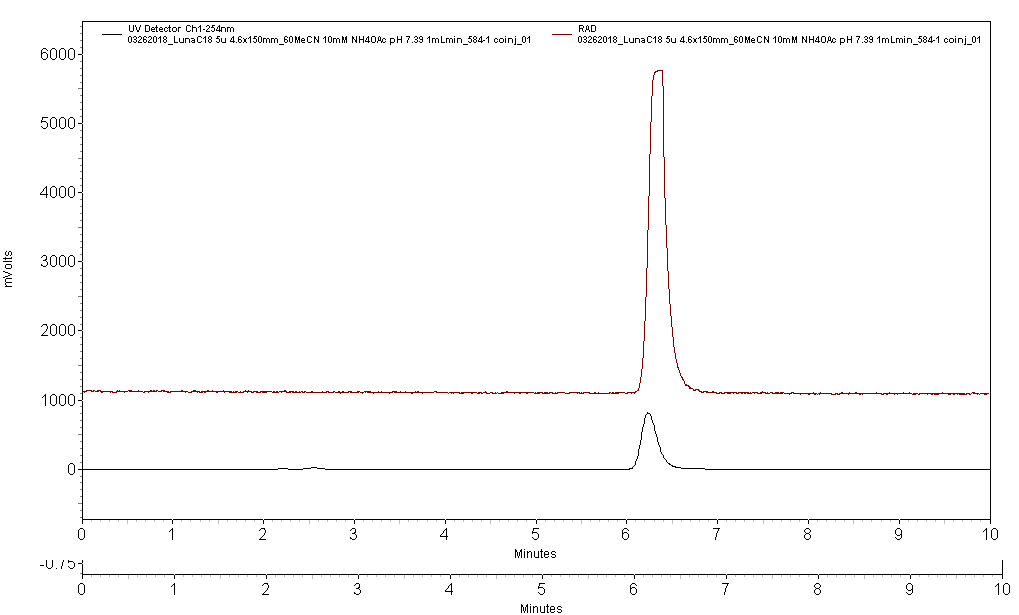


[^11^C]verubulin + genuine standard coinject QC HPLC traces (Rad above, 254 nm UV below)


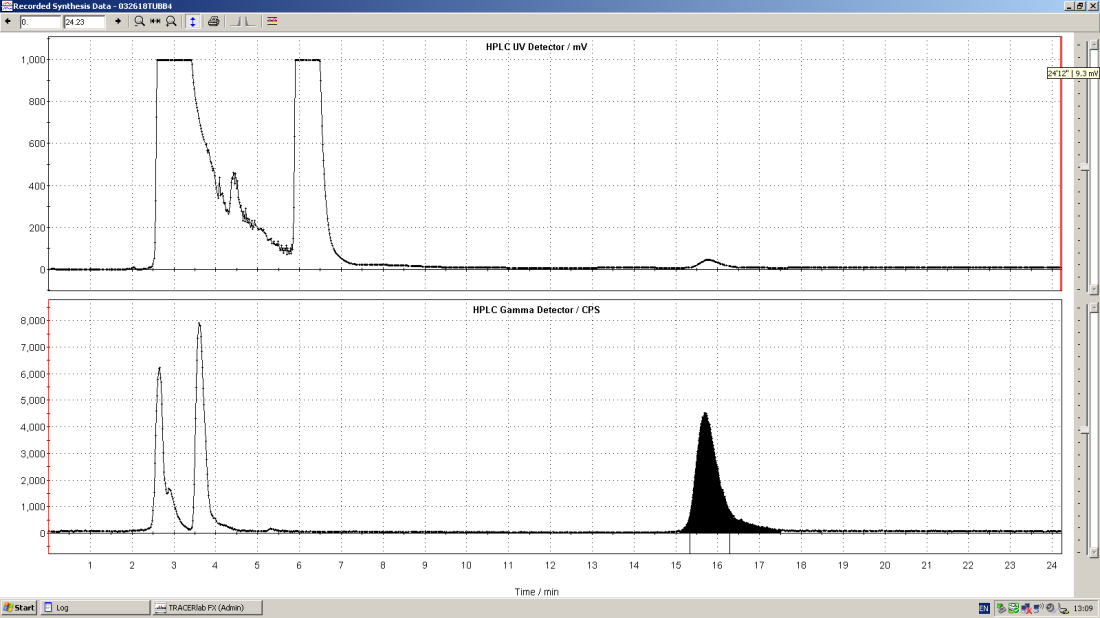


[^11^C]verubulin semipreparative HPLC traces (UV above, Rad below)

**[^11^C]HD-800**

HPLC conditions:

Semiprep column: Phenomenex Luna C18, 10 micron, 10x250 mm

Semiprep eluent: 50% MeCN 10mM NH4OAc pH:7.3 5mL/min

Analytical column: Phenomenex Luna C18, 5 micron, 4.6x150 mm, 1 mL/min

Analytical eluent: 60% MeCN 10mM NH4OAc pH:7.4 5mL/min

Delivered 30 minute beam (approx. 3 Ci [^11^C]-CO_2_) to hot cell 2. [^11^C]-CO_2_ was converted to [^11^C]-methyl iodide using standard methods; approx. 550 mCi [^11^C]-methyl iodide was produced, which was converted to [^11^C]-methyl triflate by passing gaseous through a AgOTf-on-silica column.

[^11^C]-methyl triflate was bubbled through the reactor medium (1mg precursor + 5µL 1M methanolic TBAOH in 100 µL and. DMF) at room temperature for 3 minutes at 15 mL/min with He carrier gas. Semipreparative eluent (1 mL, see above) was added to the reactor and contents of reactor were loaded onto semipreparative column. Collected peak from 11:10 - 11:55 minutes into 50mL dilution water, then passed this mixture through a Waters C18 1cc vac cartridge (pre-activated with 10 mL ethanol, then 10 mL water). Eluted product with 500 µL dehydrated ethanol into an intermediary vial, then rinsed with 4.5 mL sterile buffered saline. Passed the formulated product solution through a sterile filter and into a 10 mL product vial. Reformulated product activity: 28.7 mCi (rat). Specific activity: 2941 Ci/mmol (rat).

QC results: >99% RCP, product identity confirmed by coinjection with genuine standard.


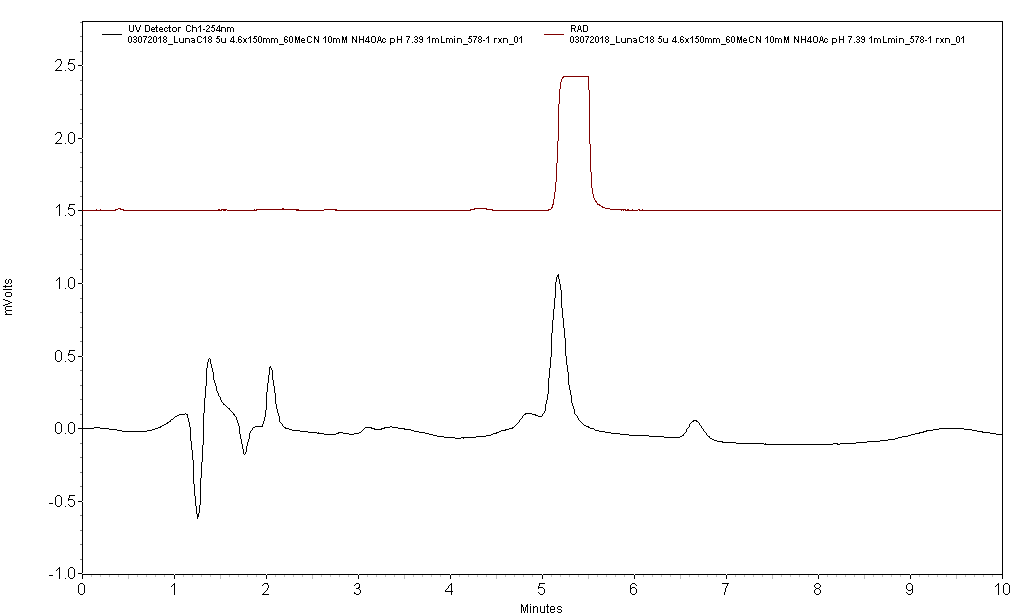


[^11^C]HD-800 QC HPLC traces (Rad above, 254 nm UV below)


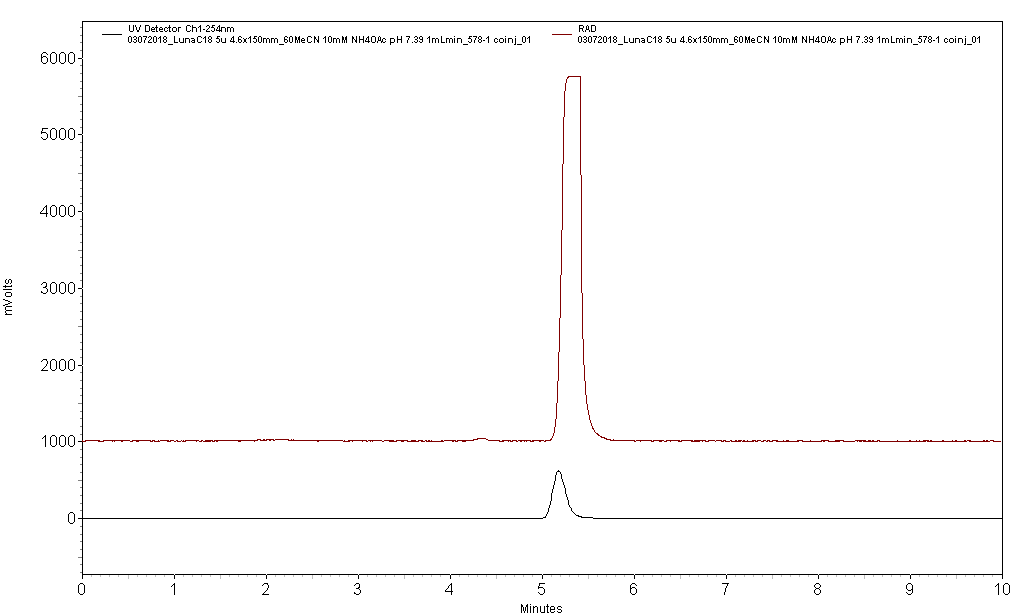


[^11^C]HD-800 + genuine standard coinjection QC HPLC traces (Rad above, 254 nm UV below)


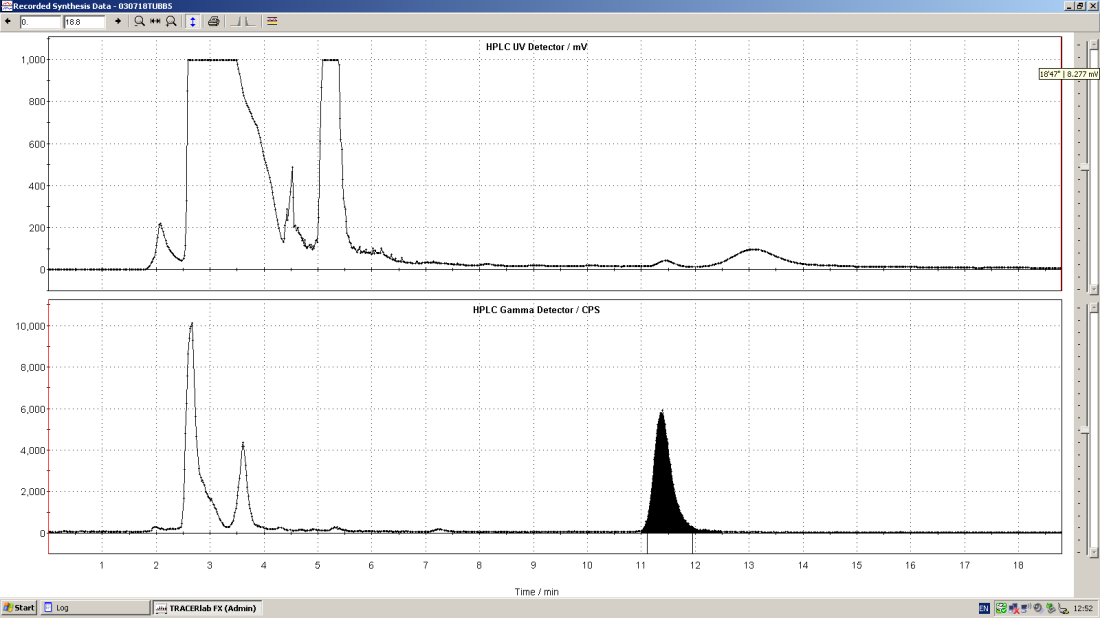


[^11^C]HD-800 semipreparative HPLC traces (UV above, Rad below)

**[^11^C]Colchicine**

HPLC conditions:

Semiprep column: Phenomenex Luna C18, 10 micron, 10x250 mm

Semiprep eluent: 50% MeCN 10mM NH4OAc pH:7.3 5mL/min

Analytical column: Phenomenex Luna C18, 5 micron, 4.6x150 mm, 1 mL/min

Analytical eluent: 60% MeCN 10mM NH4OAc pH:7.4 5mL/min

Delivered 30 minute beam (approx. 3 Ci [^11^C]-CO_2_) to hot cell 2. [^11^C]-CO_2_ was converted to [^11^C]-methyl iodide using standard methods; approx. 600 mCi [^11^C]-methyl iodide was produced, which was converted to [^11^C]-methyl triflate by passing gaseous through a AgOTf-on-silica column.

[^11^C]-methyl triflate was bubbled through the reactor medium (1mg precursor + 5µL 1M methanolic TBAOH in 100 µL and. DMF) at room temperature for 3 minutes at 15 mL/min with He carrier gas. Semipreparative eluent (1 mL, see above) was added to the reactor and contents of reactor were loaded onto semipreparative column. Collected peak from 9:10 - 9:40 minutes into 50mL dilution water, then passed this mixture through a Waters C18 1cc vac cartridge (pre-activated with 10 mL ethanol, then 10 mL water). Eluted product with 500 µL dehydrated ethanol into an intermediary vial, then rinsed with 4.5 mL sterile buffered saline. Passed the formulated product solution through a sterile filter and into a 10 mL product vial. Reformulated product activity: 13.58 mCi (rat). Specific activity: 13537 Ci/mmol (rat).

QC results: >99% RCP, product identity confirmed by coinjection with genuine standard.


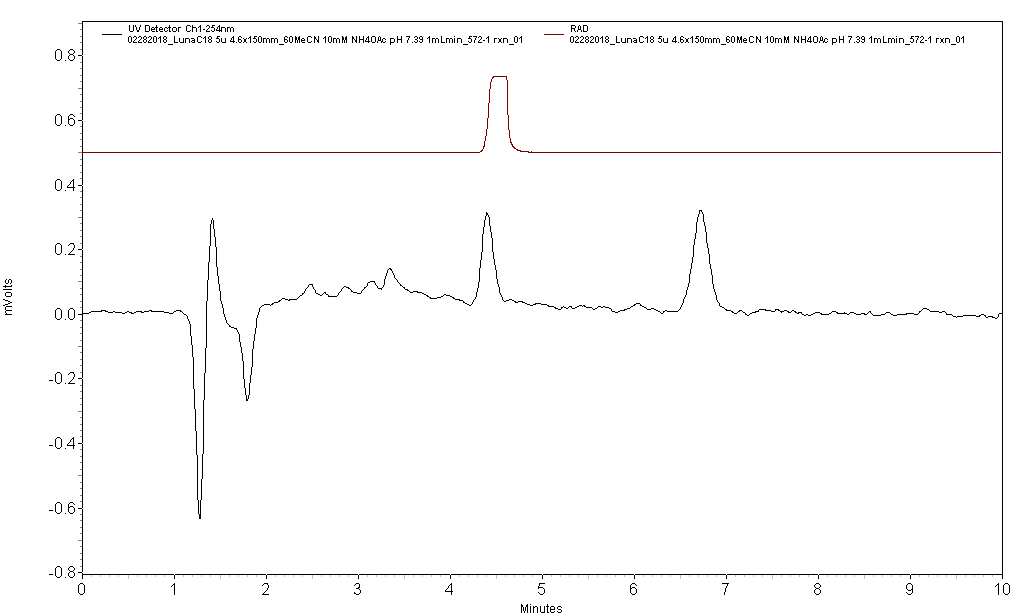


[^11^C]colchicine QC HPLC traces (Rad above, 254 nm UV below)


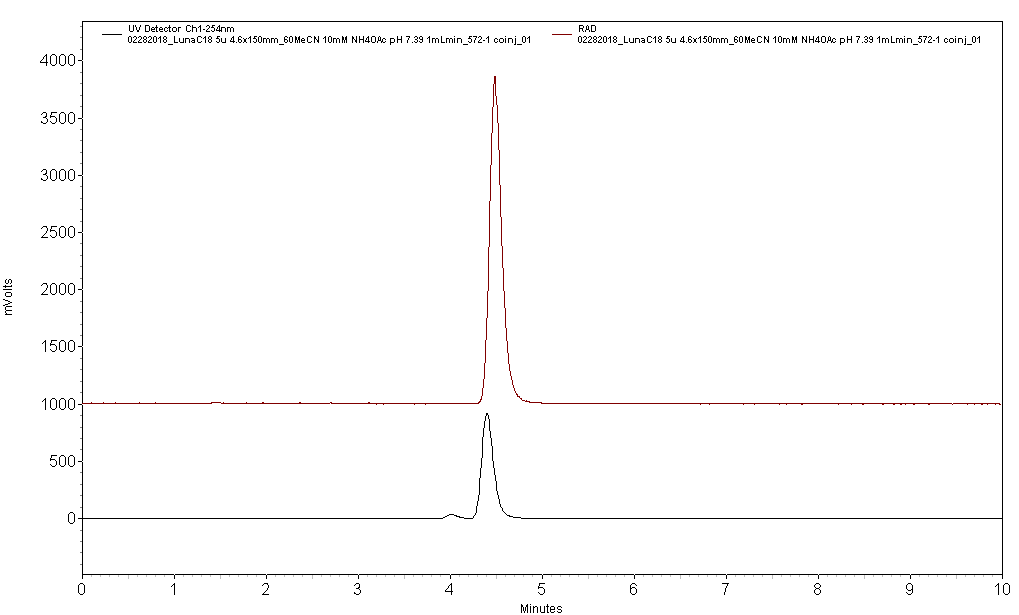


[^11^C]colchicine + genuine standard coinject QC HPLC traces (Rad above, 254 nm UV below)


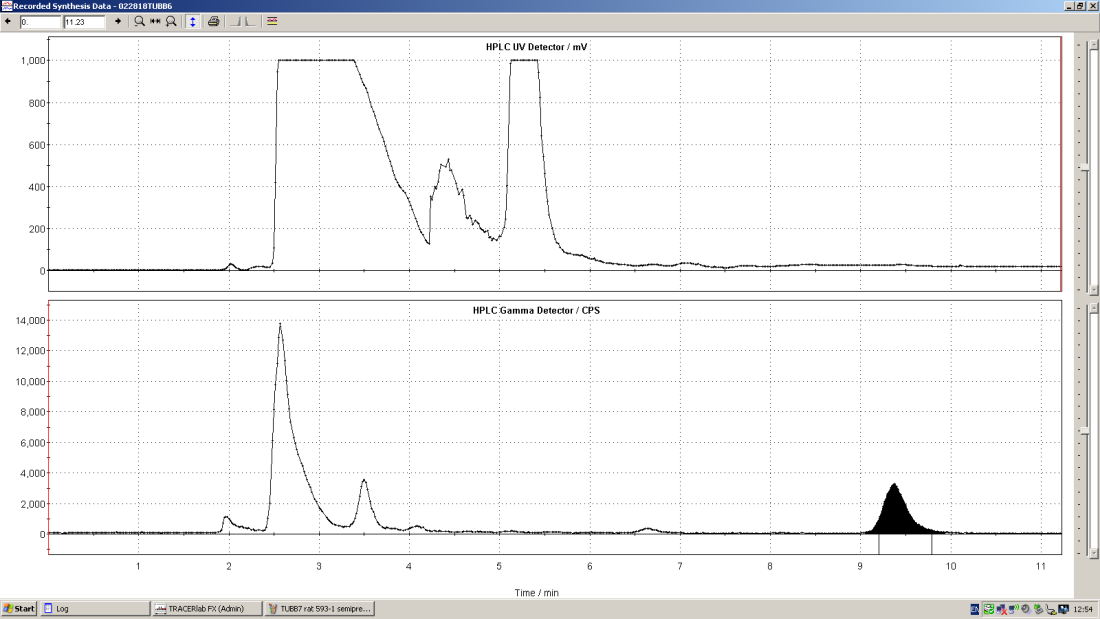


[^11^C]colchicine semipreparative HPLC traces (UV above, Rad below)

Complete list of 371 kinases tested in off target screening with [^11^C]verubulin.

|  |  |  |  |  |
| --- | --- | --- | --- | --- |
|  | **% Enzyme Activity (relative to DMSO controls)** | | **Control Compound IC50* (M):** | **Control Compound ID:** |
|  |  |  |  |  |
|  | **TUBB-4** | |  |  |
| **Kinase:** | **Data 1** | **Data 2** |  |  |
| **ABL1** | 103.50 | 102.03 | **3.84E-08** | **Staurosporine** |
| **ABL2/ARG** | 98.43 | 98.34 | **1.79E-08** | **Staurosporine** |
| **ACK1** | 90.55 | 89.15 | **1.20E-08** | **Staurosporine** |
| **AKT1** | 96.64 | 95.83 | **7.00E-09** | **Staurosporine** |
| **AKT2** | 90.66 | 90.29 | **8.15E-09** | **Staurosporine** |
| **AKT3** | 98.95 | 98.09 | **3.38E-09** | **Staurosporine** |
| **ALK** | 90.32 | 87.70 | **1.05E-09** | **Staurosporine** |
| **ALK1/ACVRL1** | 91.23 | 90.47 | **1.86E-08** | **LDN193189** |
| **ALK2/ACVR1** | 107.48 | 107.20 | **1.91E-08** | **LDN193189** |
| **ALK3/BMPR1A** | 104.78 | 99.80 | **5.27E-08** | **LDN193189** |
| **ALK4/ACVR1B** | 97.58 | 97.34 | **3.23E-07** | **LDN193189** |
| **ALK5/TGFBR1** | 104.11 | 102.55 | **3.64E-07** | **LDN193189** |
| **ALK6/BMPR1B** | 99.95 | 97.68 | **1.33E-08** | **LDN193189** |
| **ARAF** | 85.70 | 84.28 | **1.24E-08** | **GW5074** |
| **ARK5/NUAK1** | 106.28 | 100.09 | **1.21E-09** | **Staurosporine** |
| **ASK1/MAP3K5** | 100.50 | 98.75 | **2.09E-08** | **Staurosporine** |
| **Aurora A** | 111.53 | 109.75 | **8.62E-10** | **Staurosporine** |
| **Aurora B** | 99.47 | 98.15 | **2.82E-09** | **Staurosporine** |
| **Aurora C** | 99.74 | 98.99 | **4.39E-09** | **Staurosporine** |
| **AXL** | 87.46 | 87.11 | **4.01E-09** | **Staurosporine** |
| **BLK** | 89.33 | 88.41 | **1.58E-09** | **Staurosporine** |
| **BMPR2** | 117.87 | 114.81 | **2.35E-07** | **Staurosporine** |
| **BMX/ETK** | 113.29 | 110.62 | **4.40E-09** | **Staurosporine** |
| **BRAF** | 103.30 | 99.53 | **2.10E-08** | **GW5074** |
| **BRK** | 96.46 | 96.06 | **2.18E-07** | **Staurosporine** |
| **BRSK1** | 102.20 | 101.15 | **3.57E-10** | **Staurosporine** |
| **BRSK2** | 98.98 | 98.52 | **1.23E-09** | **Staurosporine** |
| **BTK** | 90.10 | 89.32 | **1.86E-08** | **Staurosporine** |
| **c-Kit** | 100.96 | 100.76 | **7.56E-08** | **Staurosporine** |
| **c-MER** | 99.72 | 99.56 | **8.43E-09** | **Staurosporine** |
| **c-MET** | 102.00 | 96.66 | **1.97E-07** | **Staurosporine** |
| **c-Src** | 102.83 | 101.26 | **2.39E-09** | **Staurosporine** |
| **CAMK1a** | 99.31 | 98.56 | **5.92E-09** | **Staurosporine** |
| **CAMK1b** | 103.72 | 102.74 | **1.52E-08** | **Staurosporine** |
| **CAMK1d** | 101.69 | 99.71 | **5.39E-10** | **Staurosporine** |
| **CAMK1g** | 99.57 | 99.11 | **6.46E-09** | **Staurosporine** |
| **CAMK2a** | 104.47 | 102.37 | **2.92E-11** | **Staurosporine** |
| **CAMK2b** | 96.51 | 96.24 | **5.22E-11** | **Staurosporine** |
| **CAMK2d** | 99.33 | 97.49 | **5.78E-11** | **Staurosporine** |
| **CAMK2g** | 97.11 | 95.47 | **2.29E-10** | **Staurosporine** |
| **CAMK4** | 103.42 | 100.94 | **2.84E-07** | **Staurosporine** |
| **CAMKK1** | 96.19 | 96.15 | **4.71E-08** | **Staurosporine** |
| **CAMKK2** | 105.15 | 99.64 | **1.59E-08** | **Staurosporine** |
| **CDC7/DBF4** | 104.10 | 100.39 | **1.76E-08** | **Staurosporine** |
| **CDK1/cyclin A** | 112.17 | 108.41 | **5.11E-09** | **Staurosporine** |
| **CDK1/cyclin B** | 100.87 | 100.72 | **3.54E-09** | **Staurosporine** |
| **CDK1/cyclin E** | 94.01 | 93.94 | **6.40E-09** | **Staurosporine** |
| **CDK14/cyclin Y (PFTK1)** | 108.78 | 100.45 | **1.75E-07** | **Staurosporine** |
| **CDK16/cyclin Y (PCTAIRE)** | 102.93 | 102.76 | **2.04E-08** | **Staurosporine** |
| **CDK17/cyclin Y (PCTK2)** | 106.71 | 106.12 | **9.83E-09** | **Staurosporine** |
| **CDK18/cyclin Y (PCTK3)** | 111.68 | 110.74 | **2.30E-08** | **Staurosporine** |
| **CDK19/cyclin C** | 94.23 | 94.14 | **8.50E-11** | **Staurosporine** |
| **CDK2/cyclin A** | 102.37 | 102.32 | **1.38E-09** | **Staurosporine** |
| **CDK2/Cyclin A1** | 90.82 | 90.61 | **1.53E-09** | **Staurosporine** |
| **CDK2/cyclin E** | 102.33 | 101.68 | **2.67E-09** | **Staurosporine** |
| **CDK2/cyclin E2** | 102.95 | 100.64 | **2.18E-09** | **Staurosporine** |
| **CDK2/cyclin O** | 104.24 | 101.96 | **1.69E-09** | **Staurosporine** |
| **CDK3/cyclin E** | 101.11 | 100.44 | **2.42E-09** | **Staurosporine** |
| **CDK3/cyclin E2** | 97.23 | 96.36 | **2.77E-09** | **Staurosporine** |
| **CDK4/cyclin D1** | 103.16 | 102.44 | **1.58E-08** | **Staurosporine** |
| **CDK4/cyclin D3** | 100.13 | 97.50 | **5.05E-08** | **Staurosporine** |
| **CDK5/p25** | 82.50 | 80.98 | **1.70E-09** | **Staurosporine** |
| **CDK5/p35** | 98.64 | 97.58 | **2.01E-09** | **Staurosporine** |
| **CDK6/cyclin D1** | 96.40 | 91.98 | **3.59E-09** | **Staurosporine** |
| **CDK6/cyclin D3** | 109.61 | 102.32 | **1.61E-08** | **Staurosporine** |
| **CDK7/cyclin H** | 103.15 | 102.84 | **3.15E-08** | **Staurosporine** |
| **CDK9/cyclin K** | 91.91 | 91.09 | **1.07E-08** | **Staurosporine** |
| **CDK9/cyclin T1** | 109.69 | 109.28 | **7.14E-09** | **Staurosporine** |
| **CDK9/cyclin T2** | 101.34 | 100.49 | **4.63E-09** | **Staurosporine** |
| **CHK1** | 98.00 | 97.31 | **4.04E-10** | **Staurosporine** |
| **CHK2** | 94.18 | 91.61 | **4.84E-09** | **Staurosporine** |
| **CK1a1** | 101.06 | 99.46 | **7.30E-06** | **Staurosporine** |
| **CK1a1L** | 97.41 | 94.57 | **2.83E-06** | **Staurosporine** |
| **CK1d** | 105.31 | 100.86 | **6.78E-07** | **D4476** |
| **CK1epsilon** | 97.60 | 97.53 | **3.70E-07** | **D4476** |
| **CK1g1** | 96.66 | 94.63 | **3.67E-06** | **Staurosporine** |
| **CK1g2** | 98.22 | 97.21 | **1.54E-06** | **Staurosporine** |
| **CK1g3** | 96.25 | 93.88 | **2.14E-06** | **Staurosporine** |
| **CK2a** | 86.32 | 84.62 | **4.57E-08** | **GW5074** |
| **CK2a2** | 80.56 | 79.55 | **4.28E-07** | **Staurosporine** |
| **CLK1** | 111.25 | 107.69 | **1.17E-08** | **Staurosporine** |
| **CLK2** | 97.19 | 92.80 | **4.96E-09** | **Staurosporine** |
| **CLK3** | 99.17 | 97.17 | **1.23E-06** | **Staurosporine** |
| **CLK4** | 91.84 | 91.01 | **2.03E-08** | **Staurosporine** |
| **COT1/MAP3K8** | 95.97 | 94.96 | **1.14E-05** | **Ro-31-8220** |
| **CSK** | 83.55 | 82.86 | **9.69E-09** | **Staurosporine** |
| **CTK/MATK** | 98.02 | 97.32 | **2.86E-07** | **Staurosporine** |
| **DAPK1** | 111.15 | 109.40 | **2.58E-08** | **Staurosporine** |
| **DAPK2** | 101.04 | 96.19 | **4.59E-09** | **Staurosporine** |
| **DCAMKL1** | 108.99 | 103.66 | **1.37E-07** | **Staurosporine** |
| **DCAMKL2** | 100.61 | 98.07 | **3.78E-08** | **Staurosporine** |
| **DDR1** | 102.76 | 98.64 | **2.62E-09** | **Staurosporine** |
| **DDR2** | 105.57 | 103.88 | **5.13E-10** | **Staurosporine** |
| **DLK/MAP3K12** | 81.69 | 77.77 | **1.48E-07** | **Staurosporine** |
| **DMPK** | 99.05 | 99.02 | **1.94E-08** | **Staurosporine** |
| **DMPK2** | 101.70 | 101.31 | **2.29E-10** | **Staurosporine** |
| **DRAK1/STK17A** | 99.85 | 99.36 | **4.08E-08** | **Staurosporine** |
| **DYRK1/DYRK1A** | 96.28 | 93.91 | **2.56E-09** | **Staurosporine** |
| **DYRK1B** | 95.94 | 94.30 | **8.60E-10** | **Staurosporine** |
| **DYRK2** | 94.52 | 93.16 | **1.46E-07** | **Staurosporine** |
| **DYRK3** | 100.42 | 100.31 | **2.61E-08** | **Staurosporine** |
| **DYRK4** | 99.35 | 97.77 | **1.28E-05** | **GW5074** |
| **EGFR** | 86.93 | 85.87 | **1.69E-07** | **Staurosporine** |
| **EPHA1** | 104.25 | 99.48 | **2.78E-07** | **Staurosporine** |
| **EPHA2** | 107.62 | 105.91 | **5.63E-08** | **Staurosporine** |
| **EPHA3** | 98.09 | 96.43 | **2.88E-08** | **Staurosporine** |
| **EPHA4** | 104.27 | 104.02 | **2.46E-08** | **Staurosporine** |
| **EPHA5** | 102.61 | 101.07 | **3.43E-08** | **Staurosporine** |
| **EPHA6** | 97.12 | 94.70 | **5.03E-08** | **Staurosporine** |
| **EPHA7** | 96.11 | 94.23 | **4.19E-08** | **Staurosporine** |
| **EPHA8** | 96.55 | 95.39 | **2.36E-07** | **Staurosporine** |
| **EPHB1** | 99.46 | 98.44 | **2.92E-08** | **Staurosporine** |
| **EPHB2** | 96.79 | 94.19 | **1.43E-07** | **Staurosporine** |
| **EPHB3** | 102.15 | 97.35 | **1.62E-06** | **Staurosporine** |
| **EPHB4** | 86.79 | 85.74 | **1.70E-07** | **Staurosporine** |
| **ERBB2/HER2** | 98.91 | 98.14 | **1.76E-06** | **Staurosporine** |
| **ERBB4/HER4** | 101.45 | 100.84 | **3.64E-07** | **Staurosporine** |
| **ERK1** | 104.10 | 98.99 | **2.01E-08** | **SCH772984** |
| **ERK2/MAPK1** | 101.08 | 100.47 | **1.68E-08** | **SCH772984** |
| **ERK5/MAPK7** | 96.35 | 94.87 | **2.39E-05** | **Staurosporine** |
| **ERK7/MAPK15** | 109.84 | 106.11 | **3.21E-09** | **Staurosporine** |
| **ERN1/IRE1** | 106.82 | 106.03 | **7.57E-08** | **Staurosporine** |
| **ERN2/IRE2** | 102.97 | 99.45 | **2.51E-08** | **Staurosporine** |
| **FAK/PTK2** | 101.94 | 101.35 | **1.44E-08** | **Staurosporine** |
| **FER** | 102.17 | 100.77 | **1.18E-09** | **Staurosporine** |
| **FES/FPS** | 110.60 | 108.81 | **1.79E-09** | **Staurosporine** |
| **FGFR1** | 77.90 | 74.46 | **9.14E-09** | **Staurosporine** |
| **FGFR2** | 107.29 | 105.81 | **5.39E-09** | **Staurosporine** |
| **FGFR3** | 98.50 | 97.50 | **1.32E-08** | **Staurosporine** |
| **FGFR4** | 101.84 | 98.28 | **1.16E-07** | **Staurosporine** |
| **FGR** | 101.93 | 97.20 | **1.07E-09** | **Staurosporine** |
| **FLT1/VEGFR1** | 100.87 | 100.78 | **2.89E-08** | **Staurosporine** |
| **FLT3** | 94.33 | 93.30 | **1.30E-09** | **Staurosporine** |
| **FLT4/VEGFR3** | 111.49 | 101.72 | **3.08E-09** | **Staurosporine** |
| **FMS** | 100.66 | 99.36 | **2.56E-09** | **Staurosporine** |
| **FRK/PTK5** | 98.86 | 97.28 | **2.14E-08** | **Staurosporine** |
| **FYN** | 101.91 | 100.43 | **1.74E-09** | **Staurosporine** |
| **GCK/MAP4K2** | 101.73 | 100.81 | **6.06E-10** | **Staurosporine** |
| **GLK/MAP4K3** | 105.54 | 103.32 | **8.50E-11** | **Staurosporine** |
| **GRK1** | 92.35 | 90.82 | **3.00E-08** | **Staurosporine** |
| **GRK2** | 104.54 | 103.31 | **9.06E-07** | **Staurosporine** |
| **GRK3** | 102.44 | 101.75 | **1.00E-06** | **Staurosporine** |
| **GRK4** | 105.40 | 103.00 | **1.27E-07** | **Staurosporine** |
| **GRK5** | 99.42 | 99.34 | **4.25E-08** | **Staurosporine** |
| **GRK6** | 103.13 | 101.10 | **3.99E-08** | **Staurosporine** |
| **GRK7** | 97.11 | 94.10 | **5.59E-09** | **Staurosporine** |
| **GSK3a** | 96.21 | 95.29 | **4.81E-09** | **Staurosporine** |
| **GSK3b** | 98.91 | 98.67 | **6.84E-09** | **Staurosporine** |
| **Haspin** | 107.53 | 102.13 | **1.70E-08** | **Staurosporine** |
| **HCK** | 110.15 | 109.00 | **1.99E-09** | **Staurosporine** |
| **HGK/MAP4K4** | 102.64 | 100.55 | **3.76E-10** | **Staurosporine** |
| **HIPK1** | 101.33 | 99.34 | **6.57E-07** | **Ro-31-8220** |
| **HIPK2** | 106.83 | 106.54 | **1.64E-06** | **Staurosporine** |
| **HIPK3** | 104.14 | 100.61 | **8.24E-07** | **Staurosporine** |
| **HIPK4** | 99.34 | 94.46 | **5.69E-07** | **Staurosporine** |
| **HPK1/MAP4K1** | 117.17 | 109.88 | **1.56E-07** | **Ro-31-8220** |
| **IGF1R** | 103.86 | 102.25 | **6.32E-08** | **Staurosporine** |
| **IKKa/CHUK** | 101.05 | 100.89 | **2.77E-07** | **Staurosporine** |
| **IKKb/IKBKB** | 97.30 | 95.92 | **2.70E-07** | **Staurosporine** |
| **IKKe/IKBKE** | 99.15 | 98.40 | **2.79E-10** | **Staurosporine** |
| **IR** | 101.92 | 101.81 | **1.87E-08** | **Staurosporine** |
| **IRAK1** | 110.04 | 109.97 | **4.78E-08** | **Staurosporine** |
| **IRAK4** | 100.69 | 96.72 | **8.82E-09** | **Staurosporine** |
| **IRR/INSRR** | 80.95 | 80.32 | **7.29E-09** | **Staurosporine** |
| **ITK** | 103.77 | 102.85 | **1.20E-08** | **Staurosporine** |
| **JAK1** | 99.79 | 99.57 | **5.62E-10** | **Staurosporine** |
| **JAK2** | 102.59 | 101.92 | **1.54E-10** | **Staurosporine** |
| **JAK3** | 97.10 | 96.45 | **7.98E-11** | **Staurosporine** |
| **JNK1** | 98.43 | 95.92 | **7.60E-07** | **Staurosporine** |
| **JNK2** | 100.82 | 98.71 | **2.34E-06** | **Staurosporine** |
| **JNK3** | 91.82 | 91.00 | **3.01E-07** | **JNKi VIII** |
| **KDR/VEGFR2** | 97.70 | 96.27 | **1.29E-08** | **Staurosporine** |
| **KHS/MAP4K5** | 92.83 | 91.65 | **7.73E-10** | **Staurosporine** |
| **KSR1** | 99.64 | 98.03 | **1.02E-05** | **Staurosporine** |
| **KSR2** | 104.94 | 104.84 | **1.20E-05** | **Staurosporine** |
| **LATS1** | 104.90 | 104.55 | **1.09E-08** | **Staurosporine** |
| **LATS2** | 91.94 | 87.77 | **1.32E-09** | **Staurosporine** |
| **LCK** | 98.21 | 97.30 | **3.73E-09** | **Staurosporine** |
| **LCK2/ICK** | 98.13 | 91.29 | **8.66E-08** | **Staurosporine** |
| **LIMK1** | 96.16 | 92.93 | **7.27E-10** | **Staurosporine** |
| **LIMK2** | 97.55 | 95.44 | **1.12E-07** | **Staurosporine** |
| **LKB1** | 116.32 | 112.60 | **7.68E-08** | **Staurosporine** |
| **LOK/STK10** | 104.91 | 102.33 | **1.08E-07** | **Ro-31-8220** |
| **LRRK2** | 91.48 | 90.99 | **3.93E-09** | **Staurosporine** |
| **LYN** | 99.48 | 98.40 | **1.43E-09** | **Staurosporine** |
| **LYN B** | 108.86 | 104.31 | **6.20E-09** | **Staurosporine** |
| **MAK** | 96.74 | 95.87 | **1.80E-08** | **Staurosporine** |
| **MAPKAPK2** | 96.80 | 96.64 | **1.01E-07** | **Staurosporine** |
| **MAPKAPK3** | 101.93 | 99.60 | **6.43E-06** | **Staurosporine** |
| **MAPKAPK5/PRAK** | 100.84 | 99.96 | **5.65E-07** | **Staurosporine** |
| **MARK1** | 103.48 | 102.46 | **1.75E-10** | **Staurosporine** |
| **MARK2/PAR-1Ba** | 98.77 | 98.26 | **6.27E-11** | **Staurosporine** |
| **MARK3** | 94.20 | 88.28 | **2.02E-10** | **Staurosporine** |
| **MARK4** | 96.54 | 94.97 | **7.29E-11** | **Staurosporine** |
| **MEK1** | 99.35 | 98.27 | **2.14E-08** | **Staurosporine** |
| **MEK2** | 104.11 | 99.76 | **6.56E-08** | **Staurosporine** |
| **MEK3** | 104.52 | 103.71 | **2.14E-08** | **Staurosporine** |
| **MEK5** | 109.38 | 101.31 | **3.43E-08** | **Staurosporine** |
| **MEKK1** | 91.13 | 84.47 | **5.49E-07** | **Staurosporine** |
| **MEKK2** | 115.90 | 110.34 | **1.31E-08** | **Staurosporine** |
| **MEKK3** | 80.45 | 78.54 | **2.94E-08** | **Staurosporine** |
| **MEKK6** | 99.83 | 97.42 | **5.25E-07** | **Staurosporine** |
| **MELK** | 88.56 | 87.48 | **4.06E-10** | **Staurosporine** |
| **MINK/MINK1** | 96.18 | 93.92 | **7.16E-10** | **Staurosporine** |
| **MKK4** | 101.77 | 97.39 | **1.76E-06** | **Staurosporine** |
| **MKK6** | 103.70 | 102.63 | **1.38E-08** | **Staurosporine** |
| **MKK7** | 103.65 | 103.38 | **1.59E-06** | **Staurosporine** |
| **MLCK/MYLK** | 103.05 | 101.64 | **3.57E-08** | **Staurosporine** |
| **MLCK2/MYLK2** | 84.69 | 83.18 | **1.25E-08** | **Staurosporine** |
| **MLK1/MAP3K9** | 95.94 | 95.44 | **9.98E-10** | **Staurosporine** |
| **MLK2/MAP3K10** | 99.86 | 94.89 | **2.96E-09** | **Staurosporine** |
| **MLK3/MAP3K11** | 101.13 | 100.30 | **2.73E-09** | **Staurosporine** |
| **MLK4** | 98.50 | 97.78 | **1.93E-06** | **Staurosporine** |
| **MNK1** | 98.29 | 95.99 | **4.88E-08** | **Staurosporine** |
| **MNK2** | 93.20 | 92.43 | **1.17E-08** | **Staurosporine** |
| **MRCKa/CDC42BPA** | 96.88 | 89.30 | **4.17E-09** | **Staurosporine** |
| **MRCKb/CDC42BPB** | 101.25 | 96.59 | **2.10E-09** | **Staurosporine** |
| **MSK1/RPS6KA5** | 100.60 | 95.00 | **4.30E-10** | **Staurosporine** |
| **MSK2/RPS6KA4** | 103.92 | 100.46 | **3.06E-09** | **Staurosporine** |
| **MSSK1/STK23** | 99.06 | 98.55 | **1.72E-06** | **Staurosporine** |
| **MST1/STK4** | 98.71 | 97.71 | **1.64E-09** | **Staurosporine** |
| **MST2/STK3** | 106.92 | 102.53 | **5.04E-09** | **Staurosporine** |
| **MST3/STK24** | 107.32 | 105.15 | **7.59E-09** | **Staurosporine** |
| **MST4** | 93.82 | 91.37 | **5.06E-09** | **Staurosporine** |
| **MUSK** | 106.32 | 104.24 | **1.64E-07** | **Staurosporine** |
| **MYLK3** | 107.38 | 106.92 | **2.08E-07** | **Staurosporine** |
| **MYLK4** | 102.32 | 101.52 | **7.00E-08** | **Staurosporine** |
| **MYO3A** | 94.48 | 93.56 | **2.60E-08** | **Staurosporine** |
| **MYO3b** | 100.31 | 99.99 | **5.82E-09** | **Staurosporine** |
| **NEK1** | 115.44 | 111.94 | **9.63E-09** | **Staurosporine** |
| **NEK11** | 93.50 | 89.83 | **1.04E-06** | **Staurosporine** |
| **NEK2** | 105.82 | 104.94 | **4.60E-07** | **Staurosporine** |
| **NEK3** | 103.03 | 100.97 | **1.99E-05** | **Staurosporine** |
| **NEK4** | 101.71 | 97.18 | **2.20E-07** | **Staurosporine** |
| **NEK5** | 89.51 | 88.07 | **4.08E-08** | **Staurosporine** |
| **NEK6** | 99.09 | 98.42 | **1.27E-05** | **PKR Inhibitor** |
| **NEK7** | 106.67 | 105.16 | **2.56E-05** | **PKR Inhibitor** |
| **NEK8** | 97.95 | 97.47 | **1.72E-08** | **Staurosporine** |
| **NEK9** | 105.46 | 104.19 | **1.14E-07** | **Staurosporine** |
| **NIM1** | 107.88 | 104.50 | **1.94E-07** | **Staurosporine** |
| **NLK** | 92.95 | 92.95 | **6.02E-08** | **Staurosporine** |
| **OSR1/OXSR1** | 106.54 | 105.08 | **1.37E-07** | **Staurosporine** |
| **P38a/MAPK14** | 101.64 | 100.02 | **2.28E-08** | **SB202190** |
| **P38b/MAPK11** | 95.29 | 92.04 | **4.61E-08** | **SB202190** |
| **P38d/MAPK13** | 100.23 | 98.19 | **1.19E-07** | **Staurosporine** |
| **P38g** | 114.18 | 112.87 | **1.23E-07** | **Staurosporine** |
| **p70S6K/RPS6KB1** | 105.79 | 105.78 | **5.34E-10** | **Staurosporine** |
| **p70S6Kb/RPS6KB2** | 106.80 | 104.10 | **1.71E-09** | **Staurosporine** |
| **PAK1** | 99.86 | 97.55 | **3.24E-10** | **Staurosporine** |
| **PAK2** | 103.64 | 100.71 | **1.77E-09** | **Staurosporine** |
| **PAK3** | 103.31 | 103.08 | **1.18E-09** | **Staurosporine** |
| **PAK4** | 107.78 | 107.00 | **2.47E-08** | **Staurosporine** |
| **PAK5** | 103.96 | 101.32 | **5.26E-09** | **Staurosporine** |
| **PAK6** | 99.72 | 99.11 | **4.50E-08** | **Staurosporine** |
| **PASK** | 94.98 | 93.82 | **1.02E-08** | **Staurosporine** |
| **PBK/TOPK** | 103.61 | 101.43 | **1.46E-07** | **Staurosporine** |
| **PDGFRa** | 98.11 | 96.51 | **9.42E-10** | **Staurosporine** |
| **PDGFRb** | 98.35 | 95.24 | **2.18E-09** | **Staurosporine** |
| **PDK1/PDPK1** | 98.73 | 98.71 | **4.90E-10** | **Staurosporine** |
| **PEAK1** | 95.28 | 95.21 | **3.98E-09** | **Staurosporine** |
| **PHKg1** | 89.65 | 89.06 | **1.85E-09** | **Staurosporine** |
| **PHKg2** | 108.74 | 106.34 | **4.76E-10** | **Staurosporine** |
| **PIM1** | 97.73 | 96.49 | **4.61E-09** | **Staurosporine** |
| **PIM2** | 95.64 | 95.36 | **2.74E-08** | **Staurosporine** |
| **PIM3** | 87.49 | 87.11 | **8.20E-11** | **Staurosporine** |
| **PKA** | 95.42 | 93.67 | **1.57E-09** | **Staurosporine** |
| **PKAcb** | 100.66 | 97.88 | **1.12E-09** | **Staurosporine** |
| **PKAcg** | 100.94 | 100.26 | **3.52E-09** | **Staurosporine** |
| **PKCa** | 99.13 | 96.91 | **6.06E-10** | **Staurosporine** |
| **PKCb1** | 108.93 | 106.28 | **3.44E-09** | **Staurosporine** |
| **PKCb2** | 102.86 | 98.63 | **1.75E-09** | **Staurosporine** |
| **PKCd** | 100.03 | 98.71 | **1.29E-10** | **Staurosporine** |
| **PKCepsilon** | 99.10 | 98.67 | **2.08E-10** | **Staurosporine** |
| **PKCeta** | 116.26 | 91.49 | **2.93E-10** | **Staurosporine** |
| **PKCg** | 112.18 | 106.21 | **1.77E-09** | **Staurosporine** |
| **PKCiota** | 108.46 | 95.94 | **1.61E-08** | **Staurosporine** |
| **PKCmu/PRKD1** | 105.99 | 101.59 | **1.17E-09** | **Staurosporine** |
| **PKCnu/PRKD3** | 97.20 | 95.26 | **9.48E-10** | **Staurosporine** |
| **PKCtheta** | 106.92 | 106.69 | **1.76E-09** | **Staurosporine** |
| **PKCzeta** | 105.84 | 105.73 | **5.59E-08** | **Staurosporine** |
| **PKD2/PRKD2** | 92.90 | 90.53 | **9.01E-10** | **Staurosporine** |
| **PKG1a** | 100.37 | 100.09 | **1.12E-09** | **Staurosporine** |
| **PKG1b** | 104.40 | 104.39 | **2.64E-09** | **Staurosporine** |
| **PKG2/PRKG2** | 88.88 | 88.76 | **1.07E-09** | **Staurosporine** |
| **PKN1/PRK1** | 96.15 | 95.74 | **1.45E-10** | **Staurosporine** |
| **PKN2/PRK2** | 101.38 | 98.85 | **2.22E-09** | **Staurosporine** |
| **PKN3/PRK3** | 93.76 | 89.17 | **9.74E-09** | **Staurosporine** |
| **PLK1** | 104.94 | 104.01 | **1.89E-07** | **Staurosporine** |
| **PLK2** | 98.89 | 98.61 | **3.00E-07** | **Staurosporine** |
| **PLK3** | 105.66 | 104.54 | **3.06E-09** | **BI2536** |
| **PLK4/SAK** | 104.86 | 101.53 | **8.22E-09** | **Staurosporine** |
| **PRKX** | 98.13 | 96.48 | **1.49E-09** | **Staurosporine** |
| **PYK2** | 104.25 | 102.31 | **6.80E-09** | **Staurosporine** |
| **RAF1** | 100.60 | 97.55 | **8.19E-09** | **GW5074** |
| **RET** | 103.57 | 102.03 | **2.83E-09** | **Staurosporine** |
| **RIPK2** | 90.85 | 85.10 | **1.80E-07** | **Staurosporine** |
| **RIPK3** | 101.39 | 100.71 | **1.20E-06** | **GW5074** |
| **RIPK4** | 105.39 | 103.18 | **9.56E-07** | **Staurosporine** |
| **RIPK5** | 99.95 | 94.42 | **1.23E-07** | **Staurosporine** |
| **ROCK1** | 102.60 | 101.84 | **7.67E-10** | **Staurosporine** |
| **ROCK2** | 93.44 | 92.80 | **3.99E-10** | **Staurosporine** |
| **RON/MST1R** | 102.04 | 101.58 | **2.59E-07** | **Staurosporine** |
| **ROS/ROS1** | 94.96 | 92.58 | **2.92E-10** | **Staurosporine** |
| **RSK1** | 94.53 | 93.21 | **1.13E-10** | **Staurosporine** |
| **RSK2** | 97.01 | 94.94 | **3.35E-10** | **Staurosporine** |
| **RSK3** | 106.35 | 104.44 | **5.48E-10** | **Staurosporine** |
| **RSK4** | 95.06 | 94.07 | **7.80E-11** | **Staurosporine** |
| **SBK1** | 103.41 | 101.35 | **8.41E-08** | **Staurosporine** |
| **SGK1** | 93.87 | 91.73 | **1.14E-08** | **Staurosporine** |
| **SGK2** | 99.91 | 96.92 | **1.16E-08** | **Staurosporine** |
| **SGK3/SGKL** | 102.27 | 99.53 | **3.19E-08** | **Staurosporine** |
| **SIK1** | 106.00 | 103.88 | **8.52E-10** | **Staurosporine** |
| **SIK2** | 94.27 | 92.36 | **2.71E-10** | **Staurosporine** |
| **SIK3** | 103.62 | 103.05 | **1.09E-09** | **Staurosporine** |
| **SLK/STK2** | 96.70 | 93.39 | **1.96E-08** | **Staurosporine** |
| **SNARK/NUAK2** | 101.68 | 97.66 | **1.81E-09** | **Staurosporine** |
| **SNRK** | 98.85 | 98.00 | **1.63E-08** | **Staurosporine** |
| **SRMS** | 103.87 | 103.43 | **1.35E-05** | **Staurosporine** |
| **SRPK1** | 78.14 | 77.29 | **5.52E-08** | **Staurosporine** |
| **SRPK2** | 105.46 | 99.78 | **2.56E-07** | **Staurosporine** |
| **SSTK/TSSK6** | 113.10 | 110.33 | **2.24E-07** | **Staurosporine** |
| **STK16** | 100.38 | 93.90 | **1.91E-07** | **Staurosporine** |
| **STK21/CIT** | 105.05 | 103.26 | **6.01E-07** | **Staurosporine** |
| **STK22D/TSSK1** | 100.18 | 98.88 | **6.29E-11** | **Staurosporine** |
| **STK25/YSK1** | 100.49 | 96.02 | **3.12E-09** | **Staurosporine** |
| **STK32B/YANK2** | 106.29 | 106.07 | **3.76E-08** | **Staurosporine** |
| **STK32C/YANK3** | 95.16 | 93.51 | **2.71E-07** | **Staurosporine** |
| **STK33** | 100.55 | 100.42 | **2.44E-08** | **Staurosporine** |
| **STK38/NDR1** | 100.14 | 97.35 | **7.46E-10** | **Staurosporine** |
| **STK38L/NDR2** | 95.19 | 94.91 | **1.23E-09** | **Staurosporine** |
| **STK39/STLK3** | 100.43 | 99.65 | **2.69E-08** | **Staurosporine** |
| **SYK** | 103.23 | 102.87 | **3.07E-10** | **Staurosporine** |
| **TAK1** | 99.14 | 98.11 | **4.97E-08** | **Staurosporine** |
| **TAOK1** | 99.18 | 98.97 | **7.97E-10** | **Staurosporine** |
| **TAOK2/TAO1** | 97.88 | 95.66 | **4.71E-09** | **Staurosporine** |
| **TAOK3/JIK** | 98.46 | 97.37 | **1.06E-09** | **Staurosporine** |
| **TBK1** | 97.29 | 96.37 | **1.02E-09** | **Staurosporine** |
| **TEC** | 99.03 | 98.54 | **1.13E-07** | **Staurosporine** |
| **TESK1** | 103.41 | 101.39 | **3.94E-07** | **Staurosporine** |
| **TESK2** | 109.86 | 109.79 | **1.67E-05** | **Staurosporine** |
| **TGFBR2** | 101.44 | 95.20 | **1.25E-07** | **LDN193189** |
| **TIE2/TEK** | 101.46 | 100.46 | **7.69E-08** | **Staurosporine** |
| **TLK1** | 84.34 | 82.34 | **1.70E-07** | **Staurosporine** |
| **TLK2** | 93.83 | 89.48 | **2.82E-09** | **Staurosporine** |
| **TNIK** | 103.07 | 99.76 | **5.09E-10** | **Staurosporine** |
| **TNK1** | 106.63 | 101.66 | **4.10E-09** | **Staurosporine** |
| **TRKA** | 97.20 | 93.82 | **3.22E-09** | **Staurosporine** |
| **TRKB** | 103.36 | 103.17 | **8.42E-11** | **Staurosporine** |
| **TRKC** | 104.21 | 104.03 | **9.82E-11** | **Staurosporine** |
| **TSSK2** | 102.98 | 102.84 | **8.68E-09** | **Staurosporine** |
| **TSSK3/STK22C** | 96.03 | 94.24 | **7.32E-09** | **Staurosporine** |
| **TTBK1** | 92.17 | 92.06 | **1.91E-05** | **SB202190** |
| **TTBK2** | 109.41 | 105.18 | **4.40E-06** | **SB202190** |
| **TXK** | 95.99 | 95.89 | **2.41E-08** | **Staurosporine** |
| **TYK1/LTK** | 84.48 | 78.18 | **9.89E-09** | **Staurosporine** |
| **TYK2** | 95.93 | 95.77 | **1.19E-10** | **Staurosporine** |
| **TYRO3/SKY** | 99.75 | 99.46 | **6.12E-09** | **Staurosporine** |
| **ULK1** | 90.47 | 87.96 | **8.30E-09** | **Staurosporine** |
| **ULK2** | 96.73 | 94.63 | **5.81E-09** | **Staurosporine** |
| **ULK3** | 99.27 | 98.41 | **3.70E-09** | **Staurosporine** |
| **VRK1** | 99.44 | 97.03 | **9.75E-07** | **Ro-31-8220** |
| **VRK2** | 100.64 | 100.32 | **5.00E-05** | **Ro-31-8220** |
| **WEE1** | 101.20 | 101.05 | **4.24E-08** | **Wee-1 Inhibitor** |
| **WNK1** | 106.24 | 105.73 | **2.54E-05** | **Staurosporine** |
| **WNK2** | 92.91 | 90.90 | **2.21E-06** | **Staurosporine** |
| **WNK3** | 109.63 | 109.08 | **1.62E-06** | **Wee-1 Inhibitor** |
| **YES/YES1** | 81.18 | 80.40 | **1.33E-09** | **Staurosporine** |
| **YSK4/MAP3K19** | 101.27 | 99.08 | **1.83E-08** | **Staurosporine** |
| **ZAK/MLTK** | 103.21 | 102.21 | **1.24E-06** | **GW5074** |
| **ZAP70** | 96.55 | 96.17 | **6.23E-09** | **Staurosporine** |
| **ZIPK/DAPK3** | 107.17 | 103.77 | **1.07E-08** | **Staurosporine** |
